# Ethanol extract of *Remotiflori radix* induces endoplasmic reticulum stress-mediated cell death through AMPK/mTOR signaling in human prostate cancer cells

**DOI:** 10.1038/srep08394

**Published:** 2015-02-11

**Authors:** Aeyung Kim, Minju Im, Jin Yeul Ma

**Affiliations:** 1 Korean Medicine (KM)-Based Herbal Drug Development Group, Korea Institute of Oriental Medicine (KIOM), Daejeon 305-811, Republic of Korea

## Abstract

*Remotiflori radix* is the root of *Mosidae*, which has long been used as a traditional medicine to treat chills, fever, and phlegm discharge. The ethanol extract of *Mosidae* leaves (MLE) possesses strong antioxidant and chemopreventive activities. However, the anti-cancer effects of the *Remotiflori radix* have not been examined. We used the ethanol extract of *Remotiflori radix* (ERR) and the PC-3 and DU145 prostate cancer cell lines in this study. We found that > 100 μg/mL ERR caused dose- and time-dependent cell death. Autophagic and apoptotic cell numbers increased in a dose-dependent manner as incubation time was prolonged, and LC3 punctuation, YO-PRO-1 uptake, DNA fragmentation, activation of caspases, and PARP cleavage were induced. Phosphorylation of AMPK, ULK, and p38 was increased after ERR treatment, and the level of the ER stress marker CHOP was also elevated. AMPK knockdown dramatically blocked ERR-mediated CHOP expression and cell death, suggesting that AMPK activation and ER stress play a critical role in ERR-induced cell death. Furthermore, oral administration of ERR at 50 mg/kg efficiently suppressed tumorigenic growth of PC-3 cells with no adverse effects. These results suggest that the ERR can be used as a safe and potent alternative therapy for patients with prostate cancer.

Prostate cancer (PCa) is the most commonly diagnosed solid tumor in adult males, and its incidence increases significantly as life expectancy increases^1^,^2^. Despite remarkable advances in early diagnosis and treatment, PCa remains the second-leading cause of cancer-related death in males in the US^1^,^3^. The majority of patients with localized PCa are initially treated with radiation or surgery. Androgen deprivation therapy (ADT) and bilateral orchiectomy have been used to reduce levels of androgens, which stimulate PCa to proliferate in patients in whom initial treatment is unsuccessful and the cancer has spread beyond the prostate gland^3^. Although most patients respond to ADT at the initial stage, the majority of these patients ultimately transit from androgen-dependent PCa to androgen-independent PCa (AIPCa) for which ADT is no longer effective^4^,^5^,^6^. Most AIPCa cases exhibit resistance to current chemotherapeutics, and metastatic AIPCa is closely associated with a poor prognosis with a median survival of approximately 1 year, suggesting that novel and non-toxic therapeutic approaches to AIPCa are urgently required^7^,^8^.

Chemopreventive and chemotherapeutic interventions with naturally occurring botanicals provide a new means of managing AIPCa^9^,^10^. PC-SPES (BotanicLab, Inc., Brea, CA, USA), a proprietary combination of one American and seven Chinese herbs, induces significant dose-dependent decreased viability in androgen-dependent (LNCaP) and androgen-independent (PC-3 and DU145) human PCa cell lines^11^. Furthermore, a retrospective analysis of patients with progressing PCa despite ADT revealed that PC-SPES has measurable effects on the post-therapy decline in serum prostate-specific antigen (PSA), suggesting that PC-SPES may be an effective treatment for AIPCa, although additional study is needed to identify the active components^12^,^13^. In addition, TBS-101, a proprietary blend of six botanicals (Titan Biosciences, Mountain View, CA, USA), has potent inhibitory activity against growth and invasion of hormone-refractory and aggressive PC-3 cells in a xenograft model, mediated by the effects of multiple active compounds that target diverse cellular pathways^14^.

*Remotiflori radix* is the root of the perennial herbaceous plant *Mosidae (Adenophora remotiflora)* in the *Campanulaceae* family, which inhabits Korea and Southern China. *Mosidae* has been traditionally used in Korea and China for managing chills, fever, expectoration, and phlegm discharge^15^. An ethanol extract of *Mosidae* leaves (MLE) exerts antioxidant and chemopreventive effects against mouse colonic 26-M3.1 and B16-BL6 melanoma cells^15^. *Adenophora* species are widely distributed in China, Japan, Korea, Taiwan, and Russia, and their roots have long been used as an anti-inflammatory and anti-tussive agent in traditional medicine^16^. The extract of *Adenophora* radix contains anti-obesity, anti-oxidant, anti-bacterial, and anti-cancer activities. An extract of *Adenophora triphylla* var*. japonica* inhibits *in vitro* proliferation of human Jurkat T and human ovarian carcinoma A2780 cells and suppresses *in vivo* gastric epithelial proliferation^17^,^18^. However, the anti-cancer effects of the root of *Mosidae*, *Remotiflori radix*, have not been elucidated.

In the present study, we examined the effect of an ethanol extract of *Remotiflori radix* (ERR) in terms of induction of cell death and the mechanisms underlying its chemotherapeutic activity using the PC-3 and DU145 PCa cell lines, which have hormone-independent and invasive properties. Furthermore, we investigated whether administration of the ERR suppresses PC-3 cell tumor growth in a xenograft model.

## Results

### ERR treatment decreases cell viability and G_1_ arrest in prostate cancer cells

**Figure 1:**
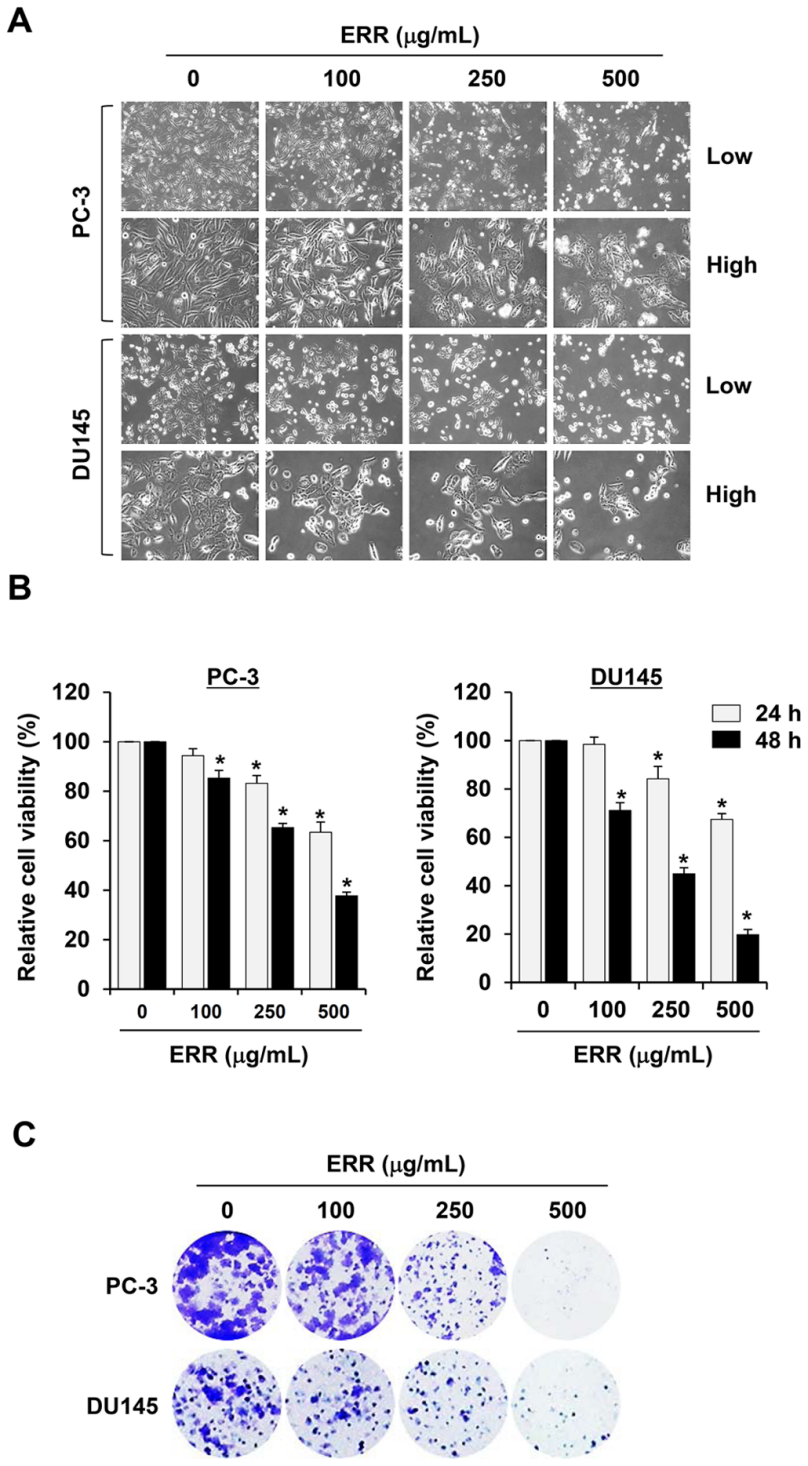
Ethanol extract of *Remotiflori radix* (ERR) induces dose- and time-dependent cell death in human prostate cancer cell lines. (A): PC-3 and DU145 cells were treated with 100, 250, and 500 μg/mL ERR for 48 h and observed under an inverted microscope (40× and 200× magnification). (B): Cell viabilities were determined by MTT assay. Data are means ± SD (n = 3) and are representative of three independent experiments carried out in triplicate. *p < 0.05 vs. untreated control. (C): At the end of incubation, anchorage-dependent colony formation was observed by staining with crystal violet.

**Figure 2:**
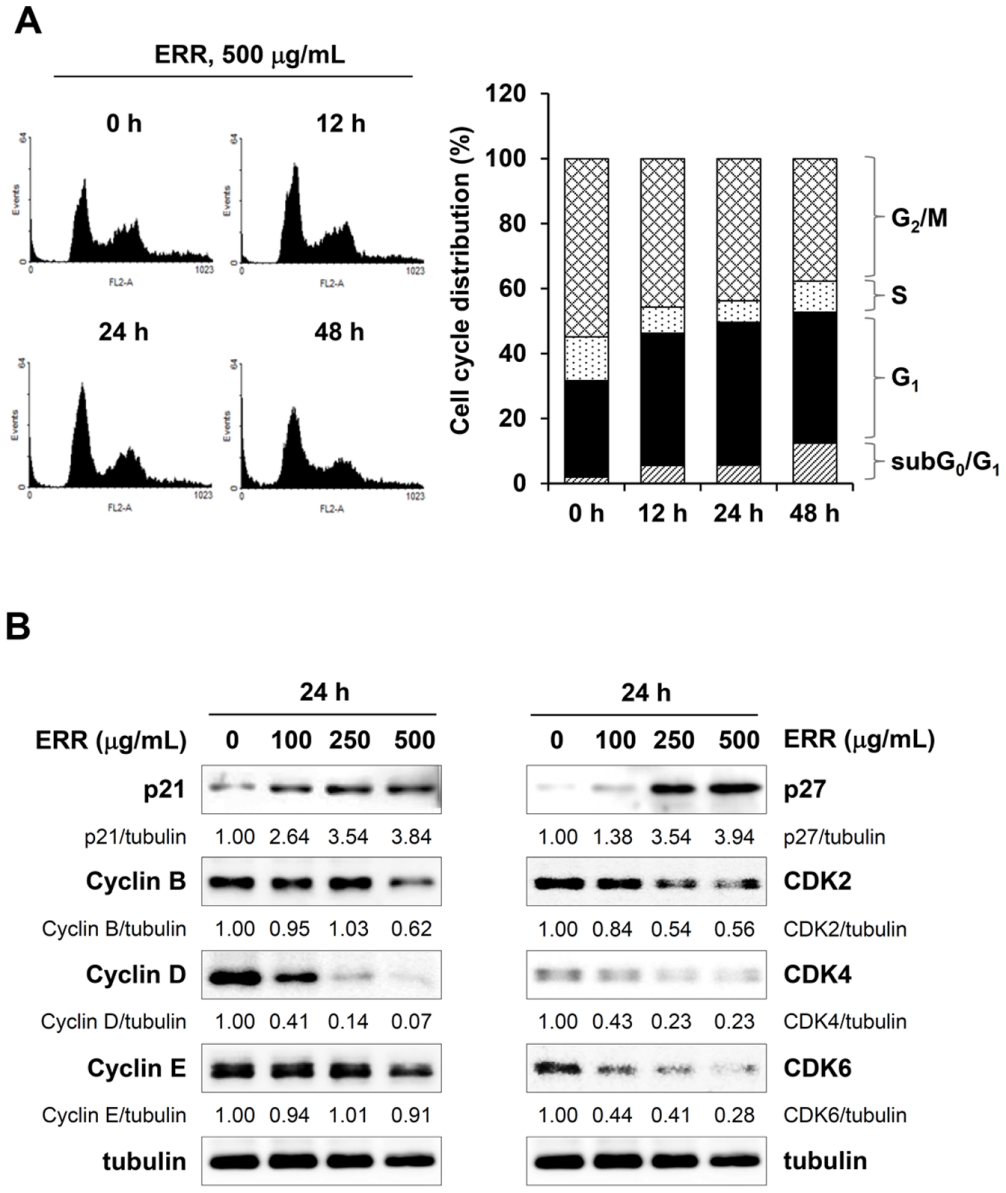
Ethanol extract of *Remotiflori radix* (ERR) arrests cell cycle progression at the G_1_ phase. (A): PC-3 cells were treated with 500 μg/mL ERR for 12, 24, and 48 h, and the cell cycle distribution was evaluated after propidium iodide (PI) staining. Data are representative of two independent experiments. (B): Expression of cell-cycle-related proteins in PC-3 cells was determined by Western blotting. Band intensities relative to those of untreated cells were calculated using ImageJ after normalization to tubulin expression. The full size blots were shown in the Supplementary Figure S1 and band of interest is indicated with an arrow.

We first assessed the morphological changes in PC-3 and DU145 cells after exposure to 100, 250, and 500 μg/mL ERR for 48 h. As shown in Figure 1A, the ERR treatment induced the majority of cells to shrink, float, and exhibit many cytoplasmic vacuoles, which is a typical apoptotic and autophagic appearance. MTT analyses showed that exposure to the ERR caused a marked decrease in cell viability in a concentration- and time-dependent manner in both cell types (Figure 1B). In addition, ERR treatment during incubation suppressed anchorage-dependent colony forming activity in a dose-dependent manner, reducing the number of sizable colonies (Figure 1C). PI staining for PC-3 cell cycle progression showed that ERR treatment for 12 and 24 h increased the proportion of cells in the G_1_ phase to 40.71 and 43.99%, respectively, compared to that in control cells (0 h, 29.65%) (Figure 2A); this increase was accompanied by a decrease in the proportions of cells in the S and G_2_/M phases. The proportions of apoptotic cells in the subG_0_/G_1_ peak increased significantly to 5.66 and 12.45% following ERR treatment for 24 and 48 h, respectively, compared to that in control cells (2.03%), indicating that ERR-mediated G_1_ cell cycle arrest prevented rapid cell proliferation and induced cell death. Western blotting showed that the ERR treatment elevated levels of the CDK inhibitors p21 and p27 in a dose-dependent manner and decreased levels of cyclin B, cyclin D, CDK2, CDK4, and CDK6 in PC-3 cells compared with those in untreated control cells (Figure 2B).

### ERR treatment causes both autophagy and apoptosis in PC-3 cells

**Figure 3:**
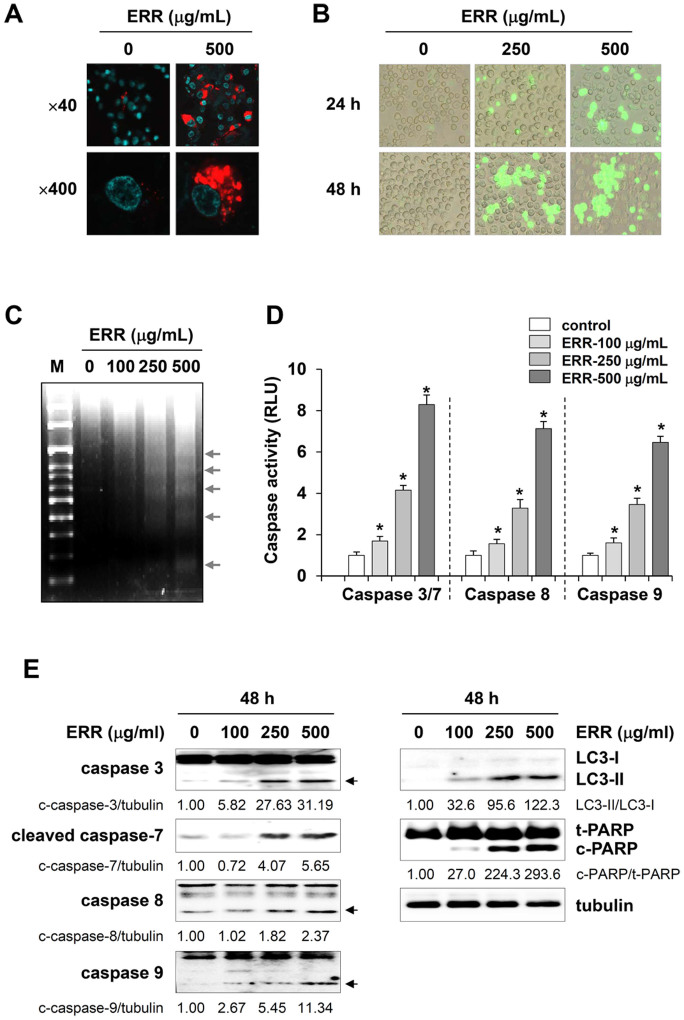
Ethanol extract of *Remotiflori radix* (ERR) induces autophagy and apoptosis. (A): PC-3 cells transiently transfected with RFP-LC3 were treated with 500 μg/mL ERR for 24 h and then observed under a confocal microscope. (B): YO-PRO-1 uptake in apoptotic cells was observed under fluorescence microscope after incubating PC-3 cells with 250 or 500 μg/mL ERR for 24 and 48 h. (C): PC-3 cells were treated with 100, 250, and 500 μg/mL ERR for 48 h and harvested. Fragmented DNA was extracted and analyzed by 1.8% agarose gel electrophoresis. (D): Caspase activities were quantified in PC-3 cells using the Caspase-Glo assay following treatment with the indicated ERR concentrations for 48 h. Luminescence signals were normalized to cell viability. Data are means ± SD of two independent experiments performed in triplicate. *p < 0.05 vs. untreated control. (E): Autophagy- and apoptosis-related markers, such as increased LC3-II, and poly ADP ribose polymerase (PARP) cleavage and increase of cleaved forms of caspases, respectively, were detected by Western blotting in ERR-treated PC-3 cells. Band intensities relative to those of untreated control cells were calculated after normalization to tubulin level. The full size blots were shown in the Supplementary Figure S3 and band of interest is indicated with an arrow.

A series of experiments was carried out to examine the cell death profile caused by the ERR. First, we assessed the distribution of LC3, an autophagy marker, in response to ERR treatment in RFP-LC3-transfected PC-3 cells. As shown in Figure 3A, control cells weakly expressed RFP-LC3 in the cytoplasm, whereas the ERR treatment markedly elevated the quantity of RFP-LC3 punctuate localization, indicating integration of LC3 into the lipid membranes of autophagosomes. Next, we assayed YO-PRO-1 uptake in ERR-treated PC-3 cells. YO-PRO-1 selectively infiltrated early stage apoptotic cells and labeled them with green fluorescence. YO-PRO-1 uptake was increased in response to the 24-h treatment with 250 and 500 μg/mL ERR compared with untreated control cells and led to a significant increase in YO-PRO-1 uptake as incubation prolonged (Figure 3B). DNA fragmentation ladders, a characteristic of apoptosis, were also observed in cells treated with ERR for 48 h (Figure 3C). Caspase-3/7, -8, and -9 activities were measured to investigate the role of caspases in ERR-induced apoptosis. As shown in Figure 3D, caspase activities were markedly enhanced in a dose-dependent manner following ERR treatment. ERR treatment also increased the levels of LC3-II, which is essential for autophagosome formation and autophagy, cleaved caspases, and PARP cleavage, in a concentration-dependent manner (Figure 3E). Two additional cell lines, DU145 and HT1080, were used to confirm the anti-cancer mechanisms of ERR. As demonstrated in the PC-3 cell system, ERR treatment decreased cell viability, caused morphological changes, suppressed colony forming activity, regulated cell cycle-related proteins, and increased LC3-II and PARP cleavage in both DU145 and HT1080 cells (Figure S2).

### ERR activates AMPK and p38 phosphorylation

**Figure 4:**
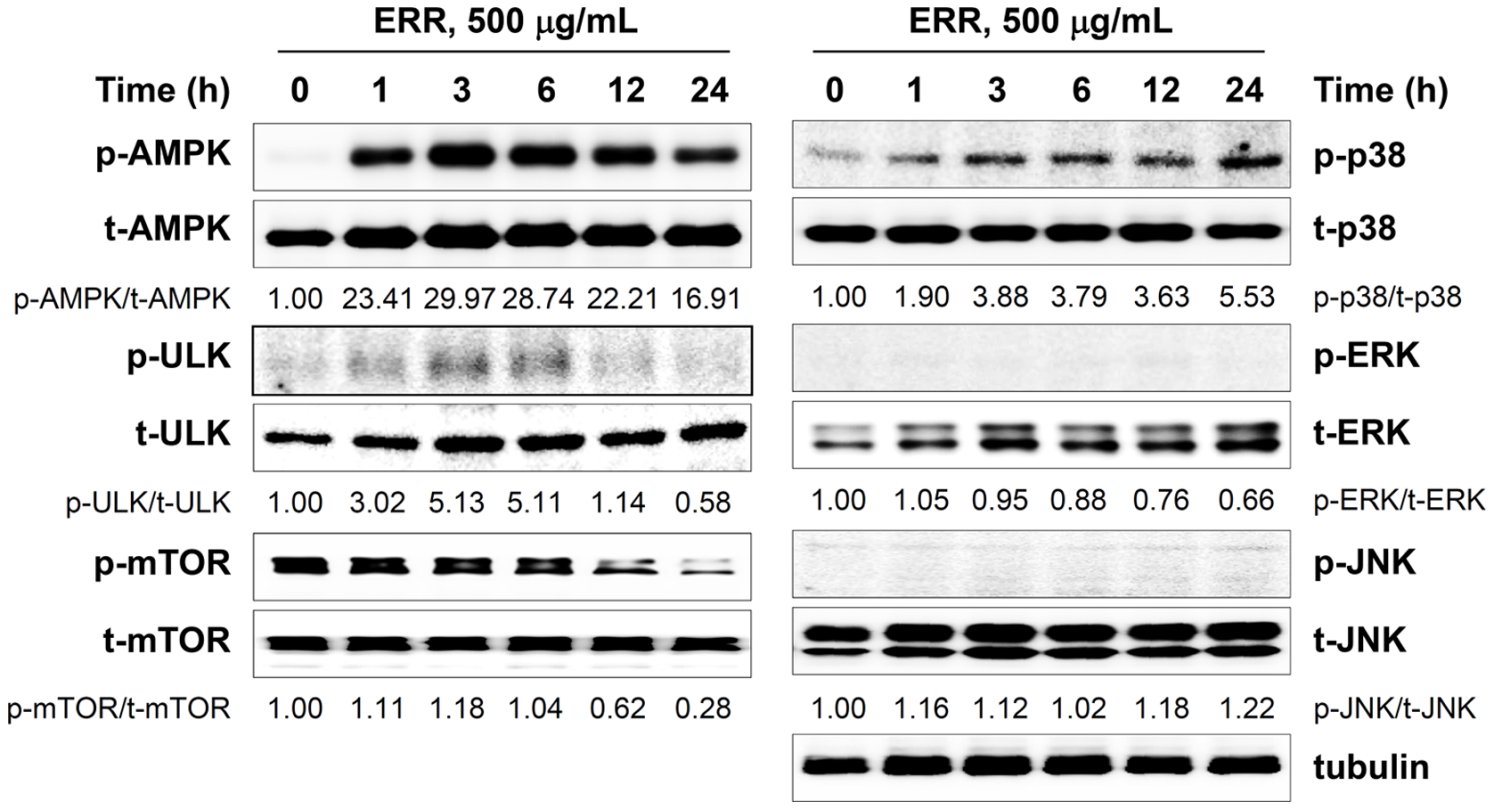
Ethanol extract of *Remotiflori radix* (ERR) activates AMP-activated protein kinase (AMPK), Unc-51-like kinase (ULK), and p38. Cell lysates prepared from PC-3 cells after treatment with 500 μg/mL ERR for the indicated periods were subjected to Western blotting. Band intensities are expressed as fold increases compared to those of untreated control cells after normalization to tubulin expression. The full size blots were shown in the Supplementary Figure S4 and band of interest is indicated with an arrow.

We examined the levels of signaling proteins critical for energy metabolism and proliferation to elucidate the mechanism of ERR-induced cell death. AMPK, a heterotrimeric complex composed of a catalytic α-subunit and two regulatory subunits (β and γ), is activated under metabolic stress and can activate the ULK1 protein kinase to induce cell death by inhibiting mTORC1 activity^19^,^20^. Western blot analyses revealed that ERR treatment significantly increased phosphorylated AMPK and ULK1 levels beginning 1 h post-treatment, and subsequently inhibited activation of their downstream target, mTOR (Figure 4). In addition, ERR treatment caused a marked increase in the phospho-p38 level, beginning 1 h after treatment and persisting to 24 h, but had little effect on ERK1/2 or JNK activation. Moreover, ERR-mediated activation of AMPK/ULK1, followed by inhibition of mTOR and activation of p38 was also detected in DU145 and HT1080 cells (Figure S5). These results demonstrate that the ERR induces cell death by activating the AMPK and p38 pathways and inhibiting the mTOR pathway.

### ER stress is involved in ERR-induced cell death

**Figure 5:**
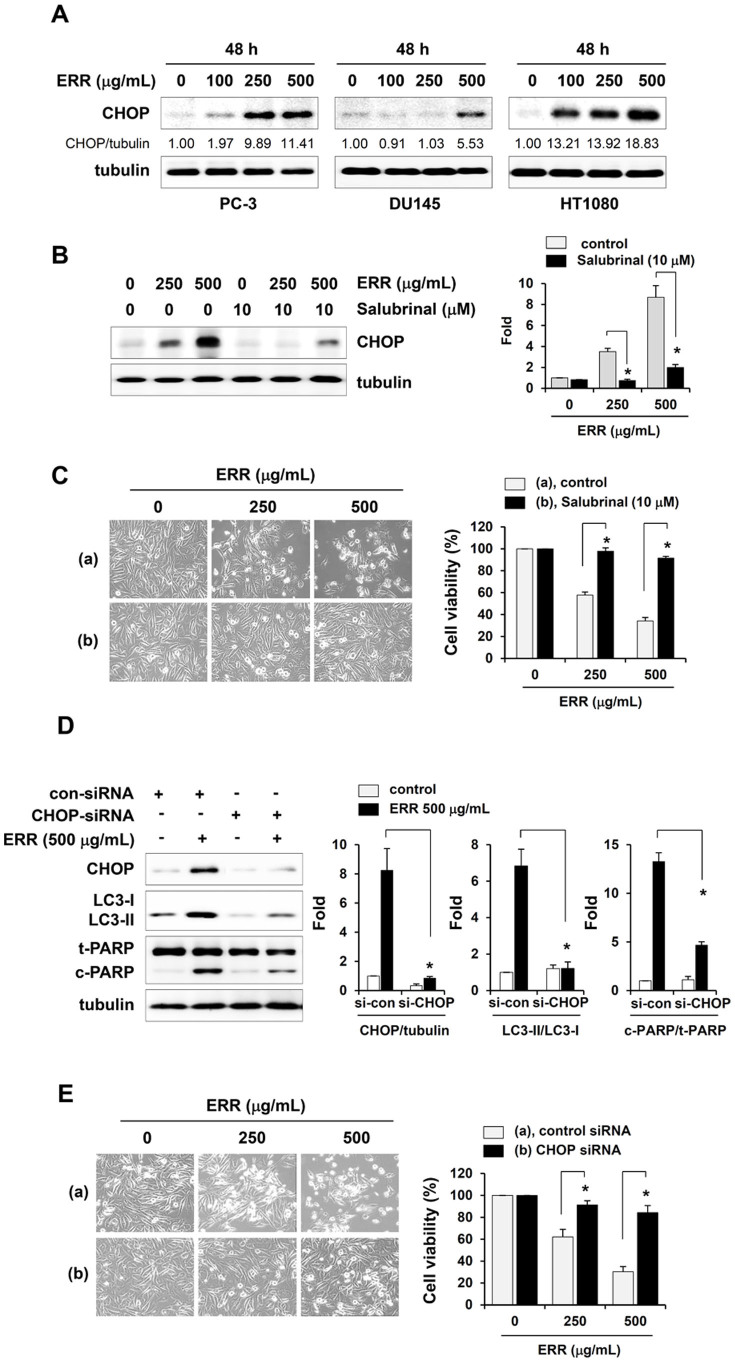
Ethanol extract of *Remotiflori radix* (ERR) induces cell death by activating endoplasmic reticulum (ER) stress. (A): CHOP levels in ERR-treated cells were detected by Western blotting. (B): PC-3 cells pretreated with or without salubrinal (1 h, 10 μM) were treated with 250 and 500 μg/mL ERR for 48 h and then examined for CHOP expression by Western blotting. (C): After treatment as described in (B), cell viability was assessed by MTT assay, and cell morphology was observed under an inverted microscope. (D): Control siRNA- or CHOP siRNA-transfected PC-3 cells were treated with 250 and 500 μg/mL ERR for 48 h and then examined for CHOP, LC3, and poly ADP ribose polymerase (PARP) expression by Western blotting. (E): Viability of cells treated as described in (D) was assessed by MTT assay, and cell morphology was observed under an inverted microscope. The fold increase relative to untreated control cells was determined after normalization to tubulin expression. Relative cell viability was quantified compared to ERR-untreated cells. Western blotting data are means ± SD of three independent experiments and MTT assay data are means ± SD of two independent experiments performed in triplicate. *p < 0.05 vs. untreated control. The full size blots were shown in the Supplementary Figure S6 and band of interest is indicated with an arrow.

It has been demonstrated that prolonged ER stress participates in the induction of autophagy and apoptosis, and could thus be responsible for the cell death induced by anti-tumor agents^21^,^22^,^23^,^24^. Due to its role as a key pro-apoptotic transcription factor during endoplasmic reticulum (ER) stress, CHOP levels were measured by Western blotting. As shown in Figure 5A, ERR treatment (48 h, 500 μg/mL) increased the CHOP level in a dose-dependent manner by ~10-, 5.5-, and 20-fold in PC-3, DU145, and HT1080 cells, respectively. We used salubrinal, a selective ER stress inhibitor, and CHOP-specific siRNA to knockdown CHOP expression to confirm its role in the regulation of cell proliferation under ERR treatment. Pretreatment with salubrinal almost completely prevented ERR-enhanced CHOP expression (Figure 5B), and prevented ~90% of ERR-treated cells from death (Figure 5C). In addition, CHOP siRNA efficiently downregulated CHOP expression, blocked the increase in LC3-II and PARP cleavage, and reduced ERR toxicity in PC-3 cells (Figure 5D and 5E). The average viability of PC-3 cells exposed to 500 μg/mL ERR for 48 h was increased from 30.3% to 84.3% after CHOP knockdown, indicating CHOP to be a critical mediator of ERR-induced cell death.

### AMPK activation is required to induce ER stress followed by cell death in response to the ERR

**Figure 6:**
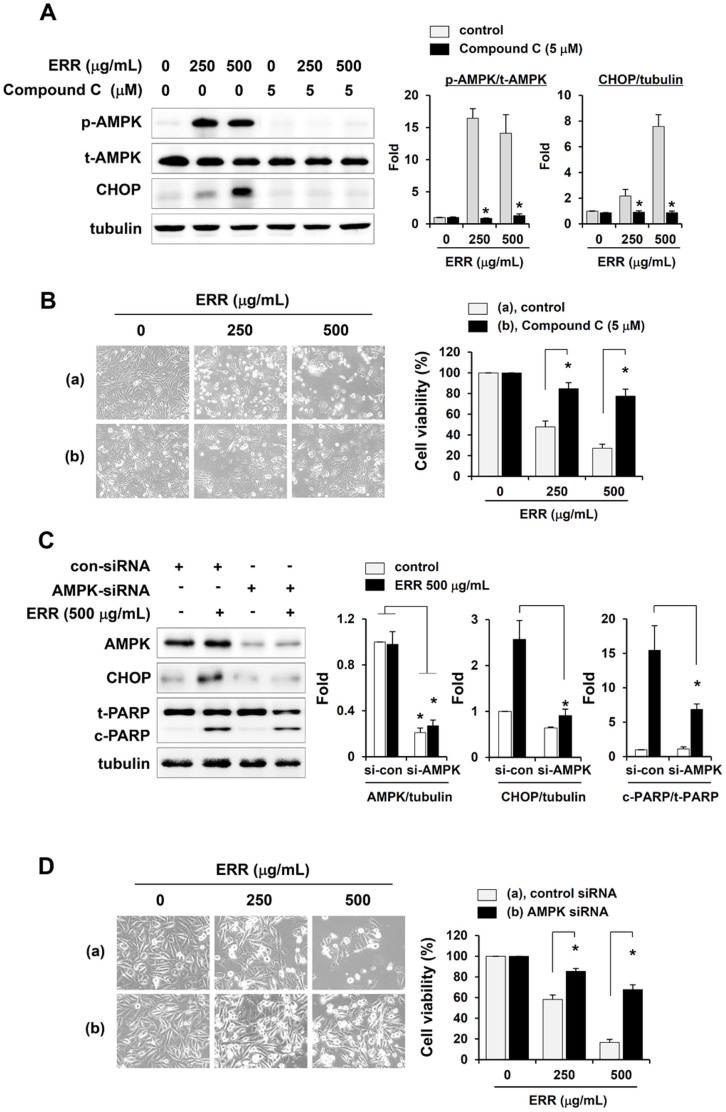
Ethanol extract of *Remotiflori radix* (ERR) induces endoplasmic reticulum (ER) stress by activating AMP-activated protein kinase (AMPK). (A): PC-3 cells pretreated with or without compound C (1 h, 5 μM) were treated with 250 or 500 μg/mL ERR for 48 h and then examined for p-AMPK and CHOP expression by Western blotting. (B): Viability of cells treated as described in (A) was assessed by MTT assay, and cell morphology was observed under an inverted microscope. (C): Control siRNA- or AMPKα1/2 siRNA-transfected PC-3 cells were treated with 250 or 500 μg/mL ERR for 48 h and then examined for CHOP and poly ADP ribose polymerase (PARP) expression by Western blotting. (D): Viability of cells treated as described in (C) was assessed by MTT assay, and cell morphology was observed under an inverted microscope. The fold increase relative to untreated control cells was determined after normalization to tubulin expression. Relative cell viability was quantified compared to ERR-untreated cells. Western blotting data are means ± SD of three independent experiments and MTT assay data are means ± SD of two independent experiments performed in triplicate. *p < 0.05 vs. untreated control. The full size blots were shown in the Supplementary Figure S7 and band of interest is indicated with an arrow.

We examined the effect of compound C, a specific inhibitor of AMPK activation, on CHOP expression in ERR-treated PC-3 cells by Western blotting to evaluate the function of AMPK in ERR-induced cell death. As shown in Figure 6A, pretreatment with compound C effectively blocked AMPK activation and completely suppressed ERR-induced CHOP expression. Furthermore, pretreatment with compound C protected ~80% of PC-3 cells from ERR-mediated cell death (Figure 6B). To verify the importance of AMPK activation in ERR-mediated cell death, we knocked down both the α1 and α2 catalytic subunits of AMPK with validated AMPK siRNA. Knock down of the α1 and α2 AMPK subunits prevented the ERR-induced CHOP expression and PARP cleavage (Figure 6C). Furthermore, AMPK knockdown rescued ERR-mediated cell death to ~85% compared to that in untreated control cells (Figure 6D). The importance of AMPK activation and CHOP expression in ERR-induced cell death was also demonstrated in HT1080 cells. Transfection with CHOP siRNA decreased ERR-induced LC3-II and PARP cleavage, and transfection with AMPK siRNA prevented ERR-induced CHOP expression and PARP cleavage in HT1080 cells (Figure S8A and S8B). In addition, knockdown of CHOP and AMPK protected ~80% of cells from ERR-induced death compared to that in untreated control cells (Figure S8C). Together, these data suggest that ERR exerts anti-cancer effects via the ER-stress- and AMPK-dependent pathways.

### ERR administration suppresses *in vivo* PC-3 cell progression

**Figure 7:**
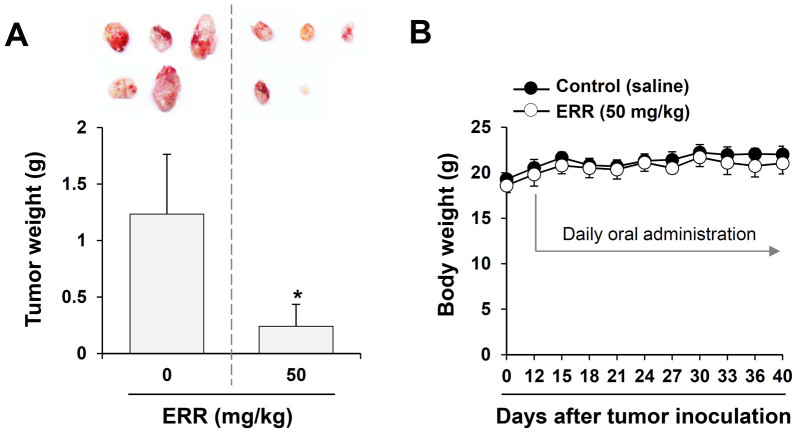
Administration of the ethanol extract of *Remotiflori radix* (ERR) suppresses *in vivo* tumor growth in a xenograft model. (A): Mice were administered daily with saline (control) or 50 mg/kg ERR for 4 weeks (n = 5 per group) 12 days after PC-3 cell (5 × 10^6^/mouse) inoculation. Final tumor weight at termination of the experiment was measured. (B): Body weights of the mice were measured every 3 days during the experiment. Data are means ± SD. *p < 0.05 vs. saline control.

Prior to determining the anti-cancer effects of ERR, we assessed the safety of the repeated administration of 50 mg/kg ERR in Balb/c nude mice. As shown in Table S1, the ratios of GOT/GPT and BUN/CRE were comparable between ERR-treated and control mice, suggesting that ERR caused no hepatic and renal dysfunction. PC-3 prostate cancer cells were injected subcutaneously into Balb/c nude mice. The mice were then administered saline (control) or 50 mg/kg ERR daily for 4 weeks commencing 12 days after tumor inoculation to assess the therapeutic efficacy of ERR *in vivo*. The masses of tumors excised from the ERR-treated mice were considerably smaller than those from control mice. Control mice exhibited a mean tumor weight of 1.234 ± 0.531 g, compared to 0.241 ± 0.194 g for mice treated with 50 mg/kg ERR; an 80% reduction in tumor weight following ERR administration (Figure 7A). In addition, no marked difference in body weight was observed between control and ERR-treated mice throughout the treatment period, indicating that ERR did not exert severe toxic effects (Figure 7B).

## Discussion

PCa is the second leading cause of cancer-related death in American males, is responsible for ~30,000 deaths per year, and has exceeded heart disease as the top killer of males > 85 years of age in the US. PCa is typically diagnosed in those > 50 years of age, and its growth and progression are relatively slow until the onset of symptoms, which include difficulties in urination and sexual dysfunction^1^,^2^. Therefore, pharmacological or nutritional interventions could enhance the quality of life of patients by delaying PCa progression. Recent studies have demonstrated that dietary agents—such as selenium, vitamins D and E, lycopene, green tea polyphenols, pomegranate, silymarin, resveratrol, indole-3-carbinol, and phytoestrogens—exert chemopreventive effects by scavenging reactive oxygen species, preventing mutagenesis, and inhibiting cell proliferation, tumor metastasis, and angiogenesis^9^,^25^,^26^,^27^,^28^. Although considerable *in vitro* data demonstrate the efficacy of these approaches, adverse effects and low bioavailability remain issues. For example, vitamin E supplementation at doses > 400 IU/day is associated with an increased risk of heart failure and an increased mortality rate^29^. Therefore, well-designed animal model studies to determine the optimal dose, route, and administration period in a single or combined approach are required to maximize the clinical usefulness of such agents. Chemoprevention as well as chemotherapeutic intervention using oriental herbal formulae—such as PC-SPES, TBS-101, and KMKKT—as well as herbal extract components suggests new approaches to the management of AIPCa^1^,^11^,^13^,^19^,^30^. Treatment with celastrol, extracted from the root bark of *Tripterygium wilfordii* Hook F., results in significant retardation of PC-3 tumor growth and induction of massive apoptosis^31^.

In this study, we found that ERR doses > 100 μg/mL suppressed cell proliferation in a dose-dependent manner, induced G_1_ cell cycle arrest, and caused cell death via autophagy and apoptosis in androgen-refractory PC-3 and DU145 cells (Figures 1,2,3). In addition, the ERR also induced ER stress, as demonstrated by upregulation of CHOP expression (Figure 5A). ER stress can trigger autophagy as well as apoptosis, ultimately leading to cell death due to a dysfunctional unfolded protein response, which is unable to restore the balance of ER stress^32^. In this study, knockdown of CHOP using CHOP-specific siRNA attenuated the ERR-induced increase in LC3-II levels and PARP cleavage, and prevented cell death. Inhibition of ER stress following pretreatment with the pharmaceutical inhibitor salubrinal also efficiently prevented ERR-induced cell death, by which the involvement of ER stress in ERR-induced cell death was confirmed (Figure 5B–E). AMPK knockdown using an AMPK-specific siRNA or inhibition of AMPK activation by a pharmaceutical inhibitor resulted in suppressed CHOP expression and cell death in response to ERR, indicating that activation of AMPK acts upstream of ER stress during ERR-induced cell death (Figure 6). The ERR treatment strongly retarded the growth of PC-3 cell tumors implanted in Balb/c nude mice with no apparent toxic effects, such as loss of body weight (Figure 7). The overall appearance of ERR-treated mice was similar to that of control mice, indicating that ERR is a safe alternative treatment for AIPCa.

*Mosidae* exerts anti-oxidant and anti-metastatic activities with no adverse effects, such as reduced body weight or piloerection. Tannins extracted from fresh leaves of *Mosidae* (ML) exhibit high DPPH (1, 1-diphenyl-2-picrylhydrazyl)-radical-scavenging activity. Saponin and inulin are the principal constituents of ML^15^. Two saponins were isolated from the roots of *Adenophora* species and exhibited cytotoxicity against the A549, AGS, and HepG2 human carcinoma cell lines^30^. Our study is the first to determine the anti-cancer effects and the underlying mechanisms of the *Mosidae* root (*Remotiflori radix*), although the active components of ERR must be isolated for clinical application.

In summary, our results demonstrate that ERR induced autophagy and apoptosis through AMPK-dependent induction of ER stress. Moreover, oral administration of ERR to mice considerably suppressed PC-3 cell tumor growth without causing adverse effects. Collectively, these results suggest that the ERR is a safe alternative therapy for prostate cancer.

## Methods

### Cell lines

Human prostate adenocarcinoma PC-3 cells (KCLB no. 21435), human prostate adenocarcinoma DU145 cells (KCLB no. 30081), and human fibrosarcoma HT1080 cells (KCLB no. 10121) were obtained from the Korean Cell Line Bank (Seoul, Korea). The cells were maintained in RPMI 1640 or DMEM (Cellgro, Manassas, VA, USA) supplemented with 10% (v/v) heat-inactivated fetal bovine serum (Cellgro) and penicillin (100 U/mL)/streptomycin (100 μg/mL) (Cellgro) at 37°C in a humidified 5% CO_2_ incubator.

### Animals

Five-week-old female Balb/c nude mice were purchased from Nara Biotech (Seoul, Korea) and maintained in a specific pathogen-free facility under constant conditions (12 h light-dark cycle at 22 ± 1°C and 55 ± 5% humidity). All animal experiments were approved by the Animal Care and Use Committee of the Korea Institute of Oriental Medicine (KIOM, Daejeon, Korea; reference number #14-040) and performed according to the guidelines of the Animal Care and Use Committee at KIOM.

### Reagents and antibodies

Compound C, salubrinal, propidium iodide (PI), and 3-(4,5-Dimethyl-2-thiazolyl)-2,5-diphenyltetrazolium bromide (MTT) were purchased from Sigma Chemical Co. (St. Louis, MO, USA). Antibodies against p21, p27, cyclin B, cyclin D, cyclin E, cyclin-dependent kinase (CDK)2, CDK4, CDK6, poly ADP ribose polymerase (PARP), caspase-3, cleaved caspase-7, caspase-8, caspase-9, AMP-activated protein kinase (AMPK), p-AMPK (Thr172), Unc-51-like kinase (ULK), p-ULK (Ser555), mTOR, p-mTOR (Ser2481), p38, p-p38 (Thr180/Tyr182), extracellular regulated kinase (ERK)1/2, p-ERK1/2 (Thr202/Tyr204), c-Jun N-terminal kinase (JNK), p-JNK (Thr183/Tyr185), and CHOP were purchased from Cell Signaling Technology (Danvers, MA, USA). Anti-microtubule-associated protein light chain 3 (LC3) and α-tubulin were obtained from Sigma Chemical Co. and Santa Cruz Biotechnology Inc. (Santa Cruz, CA, USA), respectively.

### Preparation of the ERR

Dried *R. radix* was purchased from Yeongcheon Oriental Herbal Market (Yeongcheon, Korea) and stored at the KIOM herbal bank after identity confirmation by Professor Ki Hwan Bae (College of Pharmacy, Chungnam National University, Daejeon, Korea). To prepare the ERR, dried *R. radix* (30 g) was ground into a fine powder and extracted by soaking in 300-mL 70% ethanol at 40°C for 24 h in a shaking incubator. After filtration through a testing sieve (150 μm, Retsch, Haan, Germany), the extract was evaporated on a rotary evaporator and concentrated with a freeze dryer. Freeze-dried ERR powder (50 mg) was dissolved in 1-mL 10% DMSO (v/v) and filtered through a 0.22-μm disk filter for *in vitro* experiments.

### Cell proliferation assay

The cells were plated in 96-well culture plates (5 × 10^3^/well), treated with the indicated concentrations of the ERR for 48 h, and the MTT assay was carried out as described previously^33^.

### Colony formation assay

For monolayer colony formation assay, cells seeded in a 12-well culture plate (5 × 10^2^/well) in 1 mL media were incubated to allow attachment, and then added ERR at the indicated concentrations. After incubation for 7~10 days, colonies were stained with 0.2% crystal violet/20% methanol (w/v) solution. For anchorage-independent colony formation assay, cells (5 × 10^4^) suspended in 2 mL media containing 0.3% agar, 10% FBS, and specified concentration of ERR were applied to the bottom agar containing 0.6% agar and 10% FBS. During 2 weeks of incubation, colonies in soft agar were photographed.

### Cell cycle analysis

Cells in the exponential growth phase were treated with 500 μg/mL ERR for the indicated times, harvested, washed twice with ice-cold PBS, and fixed in ice-cold 70% ethanol at −20°C for at least 24 h. The fixed cells were washed twice with ice-cold PBS, and intracellular DNA was stained with PI solution (0.1% Triton X-100, 0.1 mM EDTA, 50 μg/mL RNase A, 50 μg/mL PI in PBS) at 4°C for 30 min in the dark. The cell cycle distribution was analyzed using FACSCalibur flow cytometry (BD Biosciences, San Jose, CA, USA). Data from 10,000 cells per sample were collected and analyzed using the WinMDI 2.8 software (J. Trotter, Scripps Research Institute, La Jolla, CA, USA).

### Analysis of LC3 distribution

Cells adhering to coverslips in 24-well culture plates were transfected with RFP-tagged LC3 plasmid DNA (RFP-LC3) using the TransIT^R^-2020 transfection reagent (Mirus, Madison, WI, USA), according to the manufacturer's specifications and incubated for 24 h. After the cells were treated with 500 μg/mL ERR for 24 h, RFP-LC3 distribution was observed under a confocal laser scanning microscope (FV10i-W; Olympus Optical Co., Ltd. Tokyo, Japan) after nuclear counterstaining with DAPI using VECTASHIELD mounting media (Vector Laboratories, Burlingame, CA, USA).

### Detection of YO-PRO-1 uptake

ERR-treated cells were stained with the apoptosis-specific dye YO-PRO-1 (1 μM, Molecular Probes, Eugene, OR, USA), and YO-PRO-1 uptake was observed under fluorescence microscope as described previously to detect apoptotic cells^34^.

### DNA fragmentation assay

Genomic DNA from ERR-treated cells was prepared using the Wizard Genomic DNA Purification kit (Promega, Madison, WI) to detect oligonucleosomal DNA fragmentation, which is a hallmark of apoptosis. Purified DNA was incubated for 4 h at 37°C with 50 μg/mL RNases, electrophoresed on a 1.8% agarose gel, and visualized using GreenLight™ (BioAssay Co., Daejeon, Korea).

### Measurement of caspase activity

Caspase-3/7, -8, and -9 activities were measured with the Caspase-Glo Assay System (Promega). Following treatment with the indicated concentrations of ERR for 48 h, culture supernatants were collected by centrifugation, mixed with an equal volume of caspase-Glo substrate, and incubated at 37°C. The luminescence of each sample was measured using a luminescence microplate reader (TriStar LB 941, Berthold Technologies GmbH and Co. KG, Bad Wildbad, Germany).

### Western blot analysis

Harvested cells were washed twice with PBS and whole cell lysates were obtained using the M-PER Mammalian Protein Extraction Reagent (Thermo Scientific, Rockford, IL, USA). Protein concentrations were determined using the bicinchoninic acid kit (Sigma). An equal amount of protein was electrophoresed and immunoblotted, as reported previously^33^.

### RNA interference

Cells cultured to ~20% confluence on 60-mm culture dishes were transfected with small interfering RNA (siRNA) specific for CHOP or AMPK using the TransIT^R^-2020 transfection reagent, according to the manufacturer's instructions. After 36 h, the cells were treated with 250 and 500 μg/mL ERR for 48 h, and protein levels were analyzed by Western blotting. The CHOP siRNA sequence was 5′-AAGAACCAGCAGAGGUCACAA-3′^9^. An unrelated siRNA with a sequence of 5′-CCUACGCCACCAAUUUCGU-3′ was used as a control. AMPKα1/2 siRNA was purchased from Santa Cruz Biotechnology (sc-45312).

### *In vivo* tumor xenograft model

Female Balb/c nude mice were injected subcutaneously into the abdominal region with PC-3 cells (5 × 10^6^/mouse). The mice were randomly divided into two groups (n = 5 per group) on day 12, and administered daily saline (control) or the ERR (50 mg/kg) in a volume of 100 μL for 28 days. The mice were observed carefully in terms of gross appearance and behavior, and their body weights were measured daily. Following euthanasia of the mice by intraperitoneal injection of a mixture of Zoletil (Virbac, Magny-en-Vexin, France) and Rompun (Bayer, Seoul, Korea) (2:1, 200 μl), tumors were excised for measurement of their weight.

### Statistical analysis

Data are presented as means ± standard deviation (SD). Differences between groups were analyzed using Student's *t*-test with the SigmaPlot 8.0 software (SPSS, Inc., Chicago, IL, USA). A *p*-value < 0.05 was considered to indicate a significant difference.

## Author Contributions

A.K. and J.Y.M. conceived the research; A.K. and M.J.I. designed the project; A.K. and M.J.I. performed most of the experiments; A.K. wrote the manuscripts; all authors reviewed the manuscript.

## Supplementary Material

Supplementary Information Supplementary Information
