# Supplementary material for: Ethanol extract of Remotiflori radix induces endoplasmic reticulum stress-mediated cell death through AMPK/mTOR signaling in human prostate cancer cells
Source: Sci Rep. 2015 Feb 11;5:8394. doi: 10.1038/srep08394 (PMC4323656; doi:10.1038/srep08394)
Supplement: Supplementary Information [file srep08394-s1.pdf]

Supplementary Information

Ethanol extract of *Remotiflori radix* induces endoplasmic reticulum stress-mediated cell death through AMPK/mTOR signaling in human prostate cancer cells

AEYUNG KIM, MINJU IM and JIN YEUL MA

- Supplementary Figure S1
- Supplementary Figure S2
- Supplementary Figure S3
- Supplementary Figure S4
- Supplementary Figure S5
- Supplementary Figure S6
- Supplementary Figure S7
- Supplementary Figure S8
- Supplementary Table S1

Figure S1

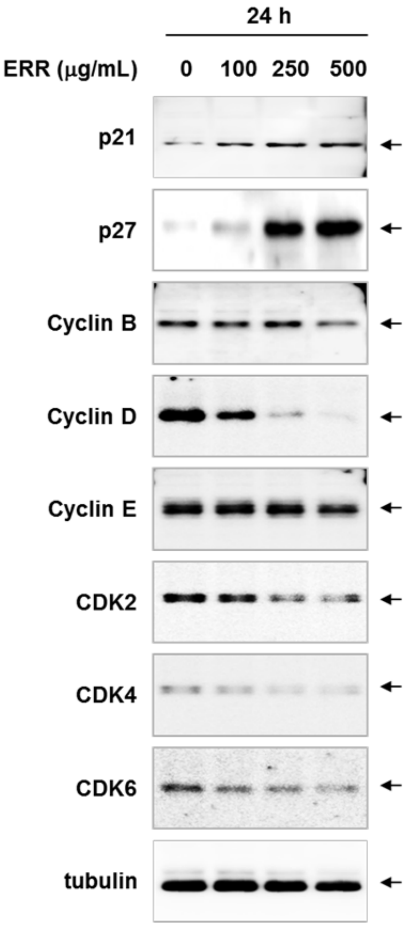

Figure S1. Expression of cell-cycle-related proteins in PC-3 cells was determined by Western blotting. This is a full length image of the cropped blot presented in the Figure 2B.

Figure S2

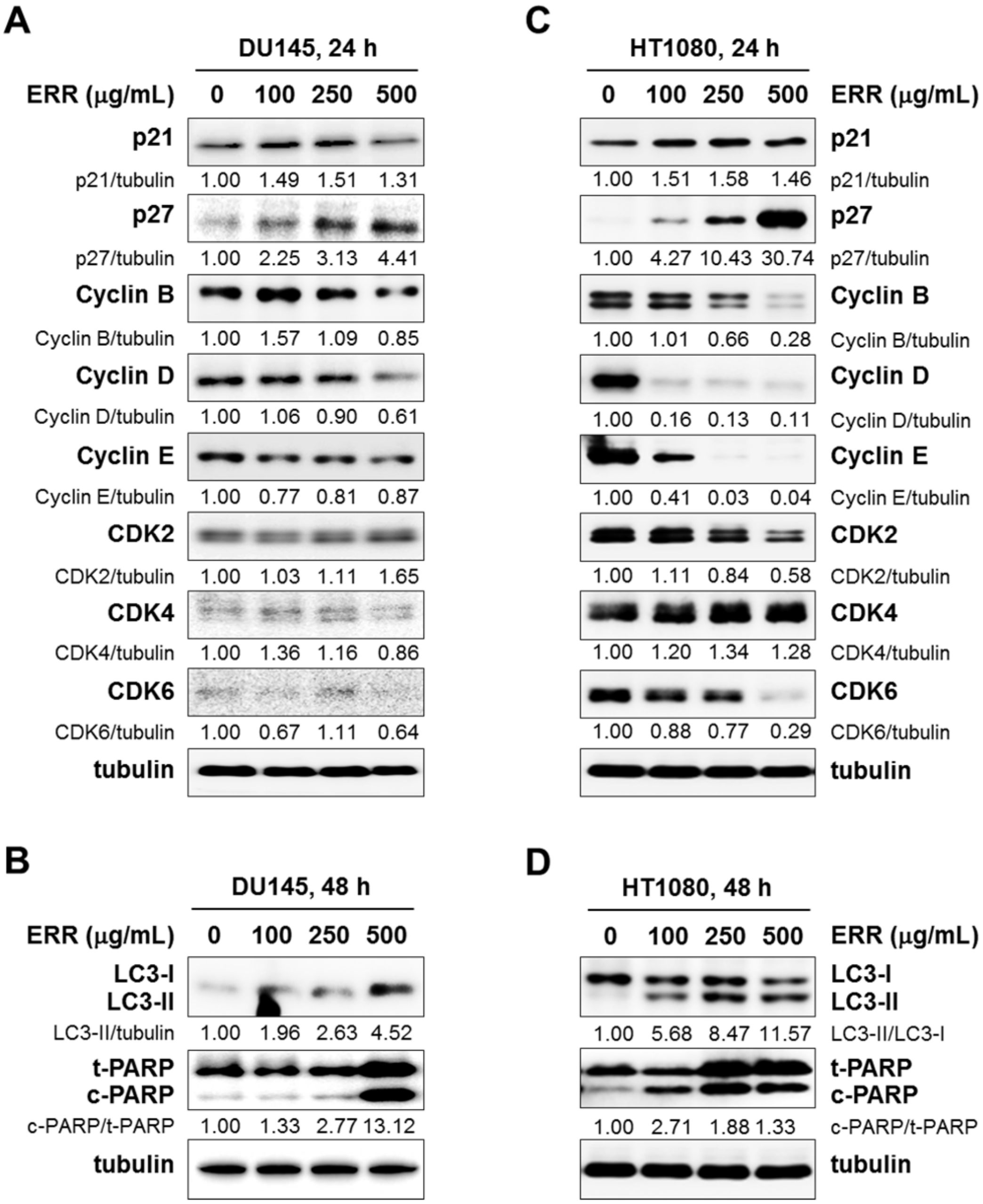
A
DU145, 24 h
ERR (μg/mL) 0 100 250 500
p21
p21/tubulin 1.00 1.49 1.51 1.31
p27
p27/tubulin 1.00 2.25 3.13 4.41
Cyclin B
Cyclin B/tubulin 1.00 1.57 1.09 0.85
Cyclin D
Cyclin D/tubulin 1.00 1.06 0.90 0.61
Cyclin E
Cyclin E/tubulin 1.00 0.77 0.81 0.87
CDK2
CDK2/tubulin 1.00 1.03 1.11 1.65
CDK4
CDK4/tubulin 1.00 1.36 1.16 0.86
CDK6
CDK6/tubulin 1.00 0.67 1.11 0.64
tubulin
C
HT1080, 24 h
0 100 250 500 ERR (μg/mL)
p21
1.00 1.51 1.58 1.46 p21/tubulin
p27
1.00 4.27 10.43 30.74 p27/tubulin
Cyclin B
1.00 1.01 0.66 0.28 Cyclin B/tubulin
Cyclin D
1.00 0.16 0.13 0.11 Cyclin D/tubulin
Cyclin E
1.00 0.41 0.03 0.04 Cyclin E/tubulin
CDK2
1.00 1.11 0.84 0.58 CDK2/tubulin
CDK4
1.00 1.20 1.34 1.28 CDK4/tubulin
CDK6
1.00 0.88 0.77 0.29 CDK6/tubulin
tubulin
B
DU145, 48 h
ERR (μg/mL) 0 100 250 500
LC3-I
LC3-II
LC3-II/tubulin 1.00 1.96 2.63 4.52
t-PARP
c-PARP
c-PARP/t-PARP 1.00 1.33 2.77 13.12
tubulin
D
HT1080, 48 h
0 100 250 500 ERR (μg/mL)
LC3-I
LC3-II
1.00 5.68 8.47 11.57 LC3-II/LC3-I
t-PARP
c-PARP
1.00 2.71 1.88 1.33 c-PARP/t-PARP
tubulin

Figure S2

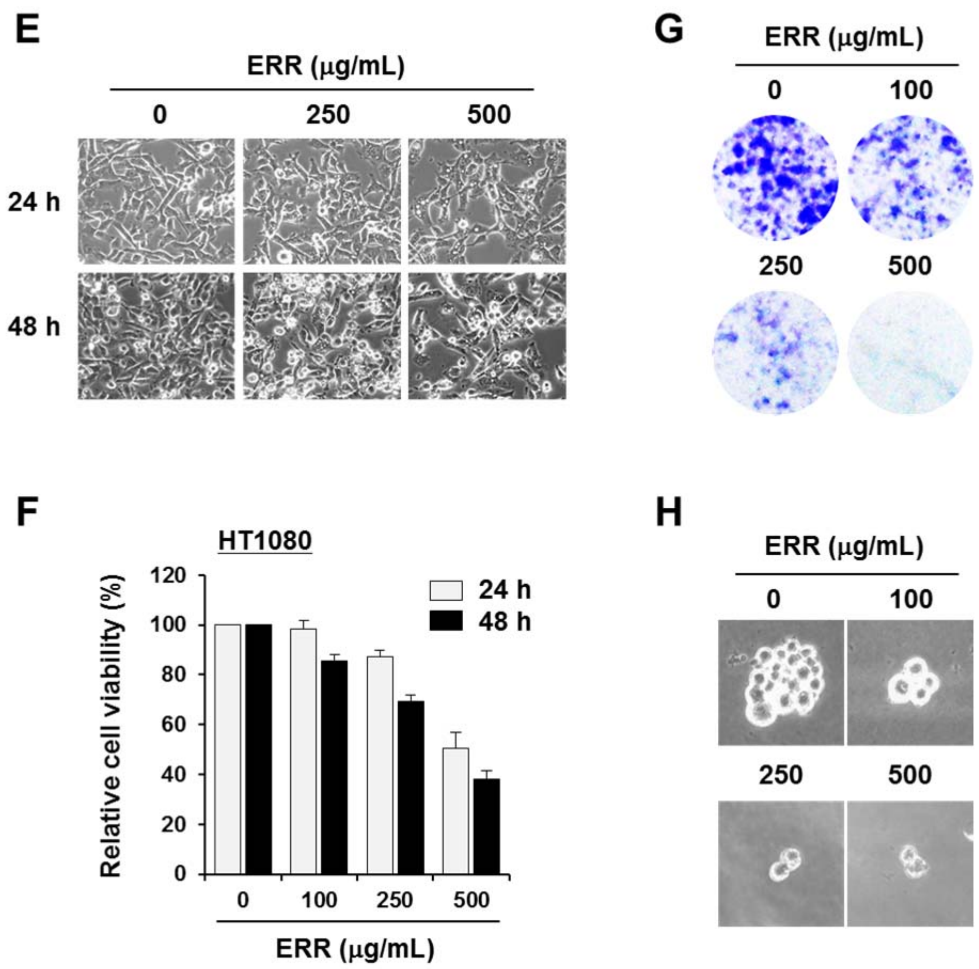

Figure S2. ERR modulates the expression of cell cycle- and cell death-related proteins in DU145 and HT1080 cells. (A-D): DU145 and HT1080 cells were treated with indicated concentrations of ERR for 24 and 48 h. Expression of specified proteins was detected by Western blotting and band intensities relative to those of untreated cells were measured using ImageJ after normalization to tubulin expression. (E and F): After treatment with ERR as indicated, cell morphology was observed under an inverted microscope and cell viability was determined by MTT assay. (G and H) Anchorage-dependent and –independent colony formation by HT1080 cells in the presence or absence of ERR were determined.

Figure S3

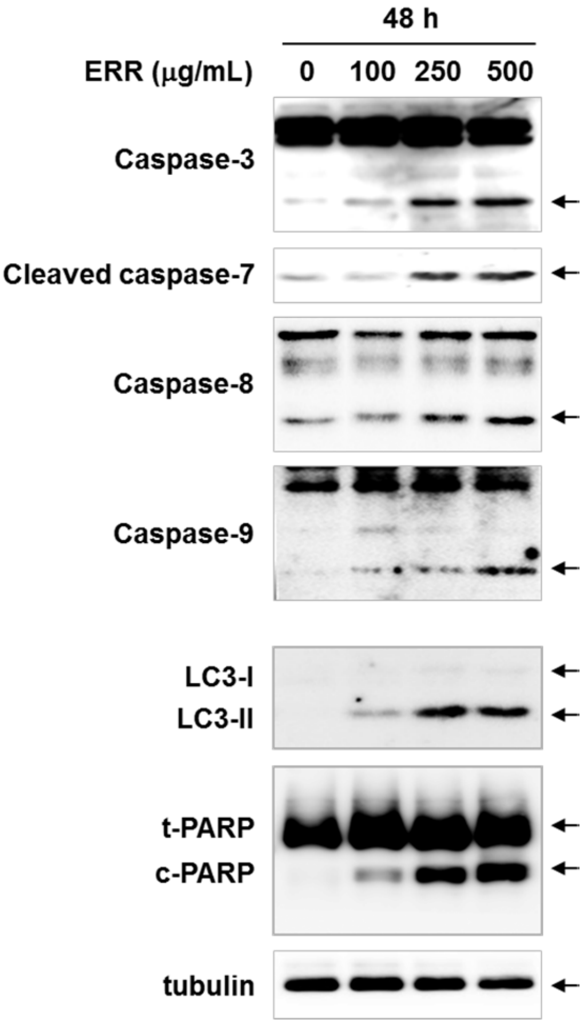

Figure S3. Expression of autophagy- and apoptosis-related proteins in PC-3 cells was determined by Western blotting. This is a full length image of the cropped blot presented in the Figure 3E.

Figure S4

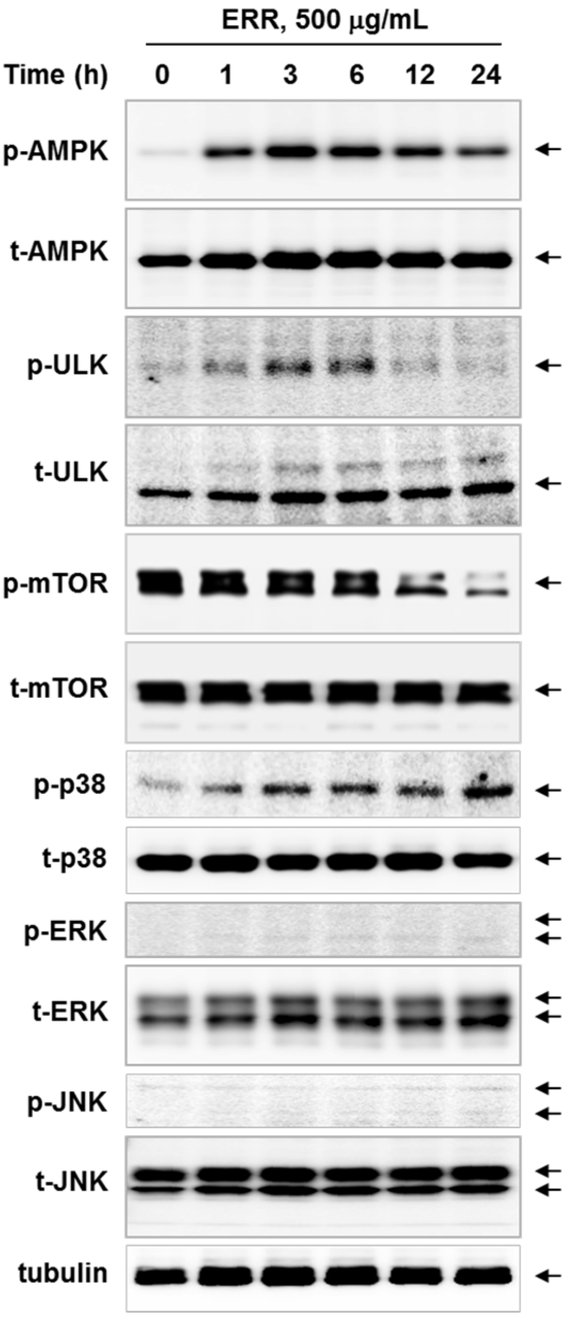

Figure S4. Ethanol extract of *Remotiflori radix* (ERR) activates AMP-activated protein kinase (AMPK), Unc-51-like kinase (ULK), and p38. This is a full length image of the cropped blot presented in the Figure 4.

Figure S5

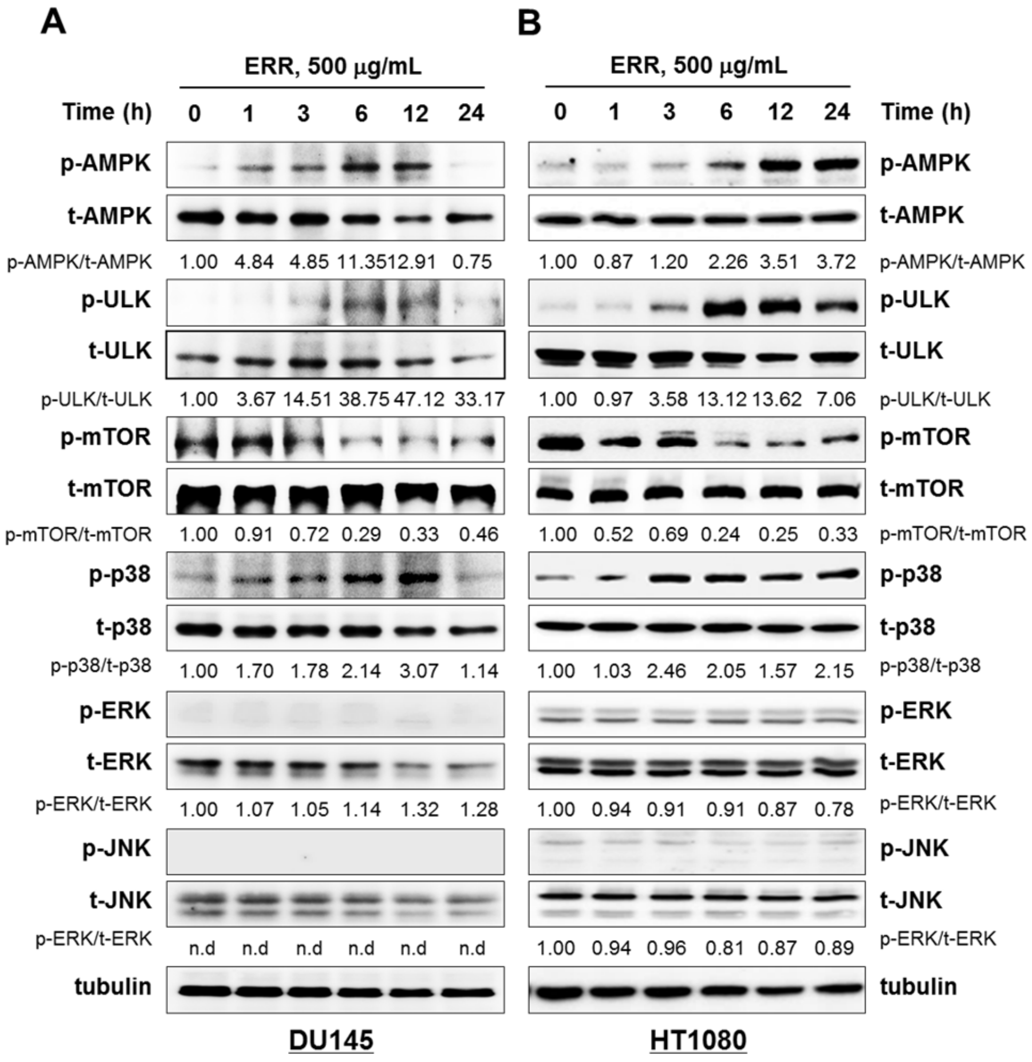

Figure S5. ERR activates AMPK, ULK, and p38 in DU145 and HT1080 cells. DU145 (A) and HT1080 (B) cells were treated with 500 μg/mL for 1, 3, 6, 12, and 24 h, and then lysates were prepared and subjected to Western blotting. Band intensities relative to those of control (0 h) were calculated using ImageJ after normalization to tubulin expression.

Figure S6

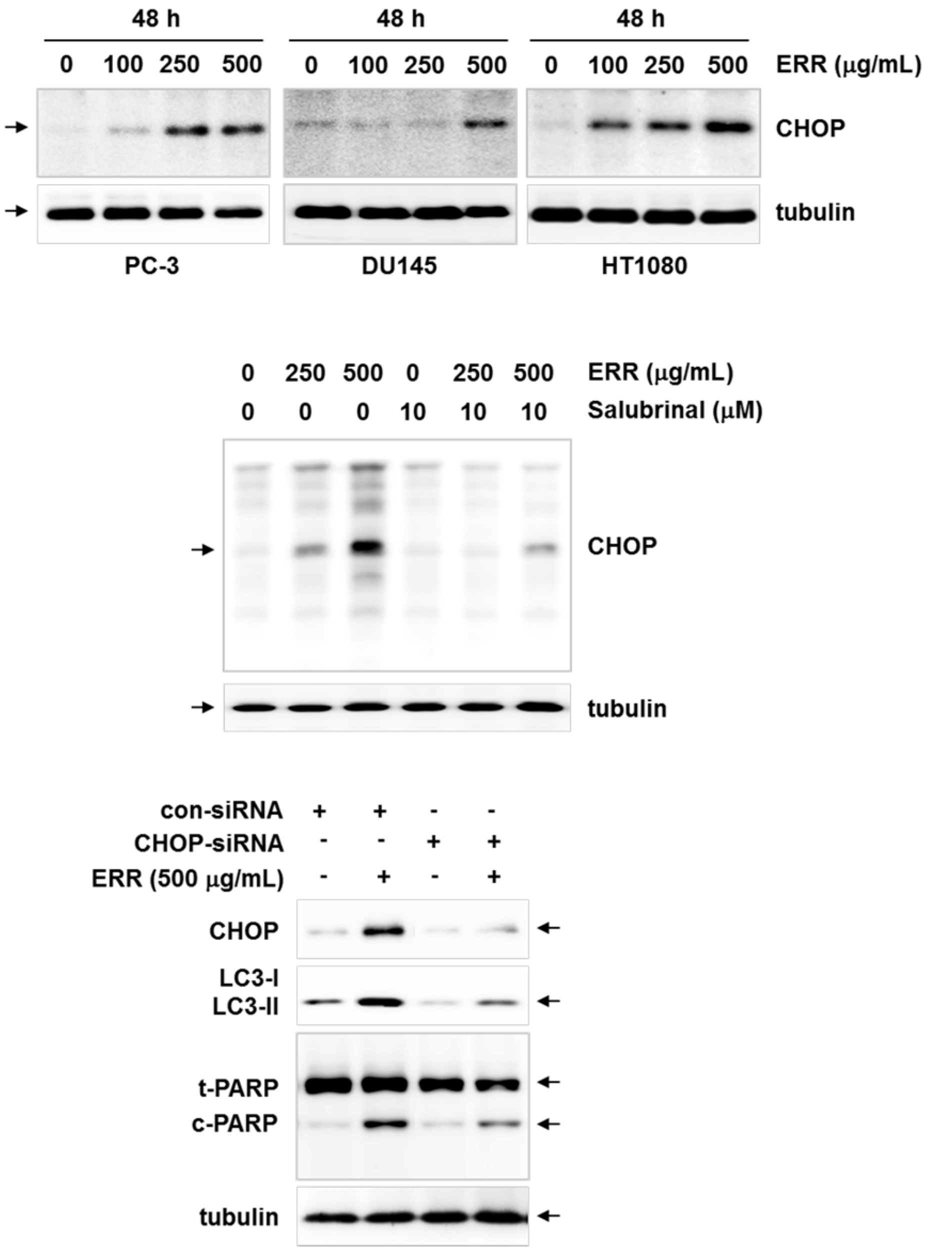

Figure S6. Ethanol extract of Remotiflori radix (ERR) induces cell death by activating endoplasmic reticulum (ER) stress. This is a full length image of the cropped blot presented in the Figure 5.

Figure S7

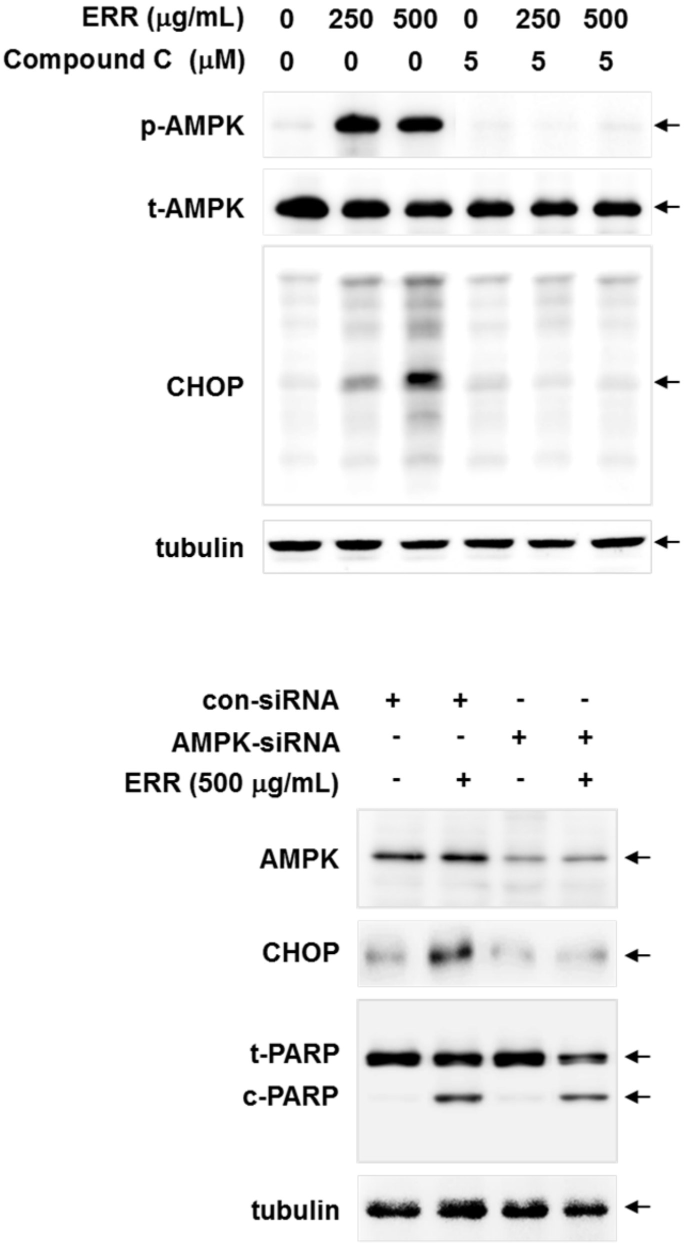

Figure S7. Ethanol extract of *Remotiflori radix* (ERR) induces endoplasmic reticulum (ER) stress by activating AMP-activated protein kinase (AMPK). This is a full length image of the cropped blot presented in the Figure 6.

Figure S8

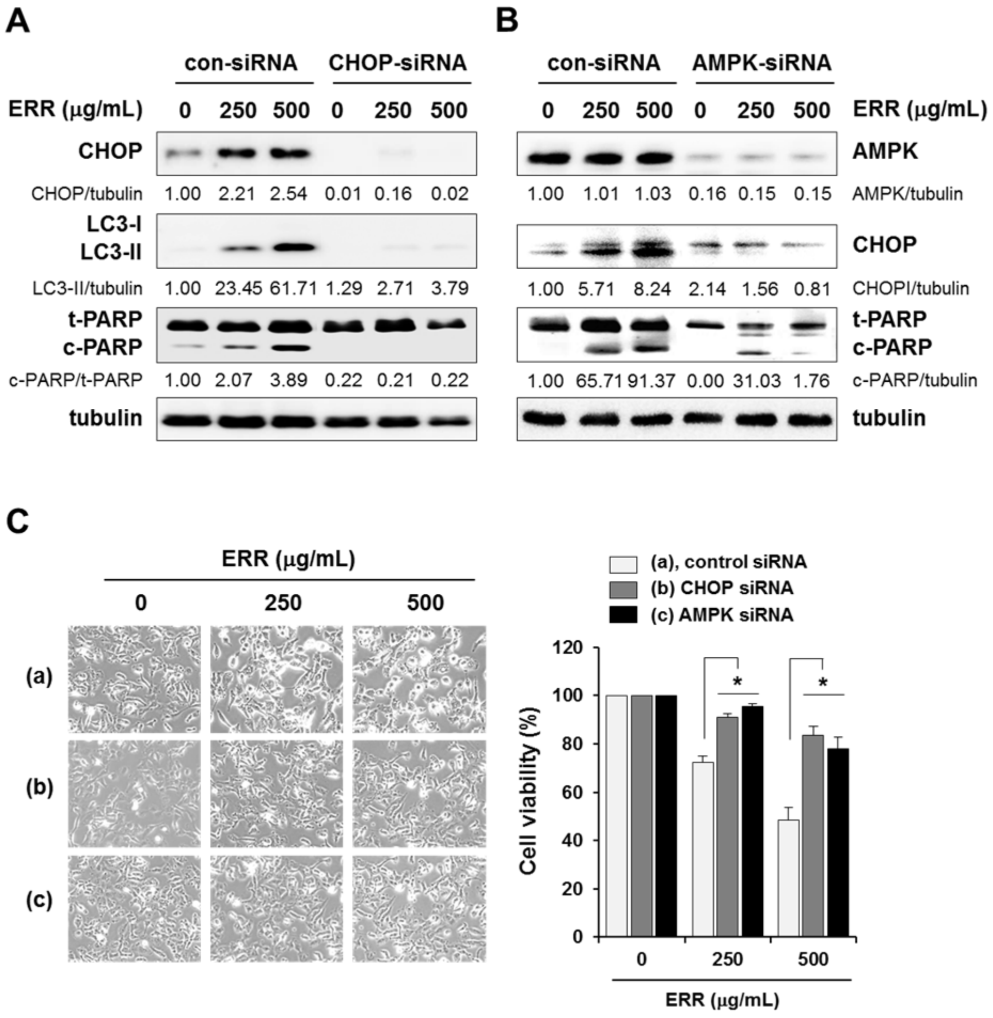

Figure S8. ERR induces cell death via AMPK-dependent ER stress in HT1080 cells. (A and B): HT1080 cells were transfected with control siRNA, CHOP siRNA, or AMPK siRNA, and then treated with ERR (250 and 500 μg/mL) for 48 h. The levels of CHOP, AMPK, LC3, and PARP were meadured by Western blotting. (C): In cells treated as described above, cell morphology was observed and cell viability was assessed by MTT assay.

Table S1

Treatment	GOT (IU/L)	GPT (IU/L)	BUN (mg/dL)	CRE (mg/dL)
control	55.21 ± 4.36	26.67 ± 3.41	25.87 ± 2.58	0.57 ± 0.21
50 mg/kg	57.38 ± 2.94	27.93 ± 1.20	23.14 ± 1.16	0.52 ± 0.05

Table S1. Safety measurement in Balb/c nude mice after oral administration of 50 mg/kg ERR. Each group of female Balb/c nude mice (n=3) were administrated with 50 mg/kg ERR or same volume of saline daily for 20 days. After sacrifice, the levels of GOT, GPT, BUN, and CRE were analyzed; GOT, glutamic oxaloacetic transaminase; GPT, glutamic pyruvic transaminase; BUN, blood urea nitrogen; CRE, creatinine. Data are presented as means ± SDs.
